# Development and validation of a prognostic nomogram for gallbladder papillary adenocarcinoma

**DOI:** 10.3389/fonc.2023.1157057

**Published:** 2023-05-16

**Authors:** Zhenfeng Wang, Longlong Wang, Yunqi Hua, Xiang Zhuang, Yu Bai, Huming Wang

**Affiliations:** ^1^ Department of Image, Baotou Cancer Hospital, Baotou, China; ^2^ Department of Surgical, Baotou Cancer Hospital, Baotou, China; ^3^ Department of Internal Medicine, Baotou Cancer Hospital, Baotou, China; ^4^ Department of Medical, West Angel Health Tech Co. Ltd., Beijing, China; ^5^ Department of Clinical Laboratory, Baotou Cancer Hospital, Baotou, China

**Keywords:** gallbladder cancer, papillary adenocarcinoma of gallbladder, propensity score matching (PSM), prognosis factors, nomogram, validation

## Abstract

**Background:**

Gallbladder papillary adenocarcinoma (GBPA) is an uncharacteristically gallbladder cancer subtype. Although some studies have shown that the prognosis of GBPA patients is significantly better than that of gallbladder adenocarcinoma (GBA) and gallbladder mucinous adenocarcinoma (GBMA) due to its rarity, there is a lack of large sample studies necessary to confirm the clinical characteristics and survival rate of GBPA. Therefore, this study aimed to describe the clinicopathological characteristics affecting survival in GBPA. This data was then used to establish a prognostic nomogram for GBPA.

**Methods:**

The data of patients diagnosed with gallbladder cancer between 2004 to 2015 were extracted from the Surveillance, Epidemiology, and End Results (SEER) database. The clinical features and survival of patients with GBPA were compared with those of GBA and GBMA after balancing the baseline characteristics using propensity score matching (PSM). Univariate and multivariate Cox analyses were used to identify the prognostic factors for GBPA. Subsequently, the overall survival (OS) and cancer-specific survival (CSS) nomograms were established to predict GBPA prognosis. The performance and discrimination of the nomogram were measured using concordance index (C-index), calibration curves, receptor operating characteristic curves(ROC), and decision curve analysis (DCA) was applied to examine the net benefit of tients with GBPA, 5798 patients with GBA, and 223 patients with GBMA. The mean 1-, 3- and 5-year OS rates for GBPA were 81.3%, 58.8%, and 49.1%, respectively, while the mean 1-, 3- and 5-year CSS rates were 85.0%, 68.1%, and 61.0%, respectively. The median OS rates was 58 months (95% CI: 43–88), while the median CSS was not reached. The PSM analysis showed a differ statistically significantly in the OS between GBPA and GBA. However, there has no statistically difference in CSS. Conversely, the OS and CSS between GBPA and GBMA have statistically significant differences. Age, marital, T stage, and M stage were strongly linked to the prognosis for OS, while T-stage, M-stage, and surgery were significantly associated with the prognosis for CSS in GBPA patients. The AUC for the 1-, 3-, and 5-year OS were 0.722 (95%CI: 0.630-0.813), 0.728 (95%CI: 0.665-0.790), and 0.706 (95%CI: 0.641-0.771), respectively. The AUC for the 1-, 3-, and 5-year CSS were 0.749 (95%CI: 0.659-0.840), 0.698 (95%CI: 0.627-0.770), and 0.665 (95%CI: 0.594-0.735), respectively. The C-indices for the OS and CSS nomograms were 0.701 (95% CI: 0.634-0.744) and 0.651 (95% CI: 0.598-0.703), respectively. The calibration curves showed that the nomograms were well consistency. The DCA showed that compared with the TNM system, the nomogram models had a significant positive net benefit in survival prediction.

**Conclusion:**

GBPA has distinct clinicopathological characteristics and survival compared to other gallbladder carcinomas. The established nomogram provided a better prediction of survival for GBPA patients than the traditional TNM models.

## Introduction

Gallbladder cancer (GBC) is the most prevalent malignant tumor of the biliary system and the sixth most prevalent malignant tumor of the digestive tract (1–3). Adenocarcinomas originating from the secretory cells of the gallbladder are the most common GBC subtype and account for more than 90% of the cases (4–6). Other rare adenocarcinomas include gallbladder papillary adenocarcinoma (GBPA), gallbladder mucinous adenocarcinoma (GBMA), and gallbladder tubular adenocarcinoma (GBTA) (4, 7). GBPA originates from the papillary cells that facilitate the movement of bile in the gallbladder. As the tumor grows, it blocks the flow of bile in the gallbladder. If the tumor blocks the neck of the gallbladder, it can lead to an enlargement of the gallbladder, thinning of the gallbladder wall, and ultimately the formation of an abscess or effusion. In addition, GBPA can also form ulcers on the tumor surface, which are prone to infection. GBPA is often found incidentally in pathological examinations of postoperative specimens. To our knowledge, few studies have been published evaluating the clinicopathological characteristics of GBPA, and most studies are limited to individual case reports or small retrospective series (8–13).

Previous studies reported that the prognosis of GBPA is generally better than the other types of GBC. Veeravich et al. (14) compared the survival of GBPA with other types of GBC using population data obtained from the Surveillance, Epidemiology, and End Results (SEER) database. The conclusion demonstrated that the survival rate of GBPA was much higher than that of GBA and other types of GBC. Although some studies (10, 15, 16) have also confirmed that the prognosis of GBPA is better than other types of GBC, these studies are more than 10 years old and did not perform a propensity score matching (PSM) analysis to evaluate the impact of other various variables on survival. Therefore this study was designed to retrospectively evaluate the clinical factors and prognosis of patients diagnosed with GBPA, applying data obtained from the SEER database. The objectives of the studies were to: (1) represent and contrast the characteristics of GBPA and other types of GBC (GBA, GBMA); (2) perform a propensity score matching (PSM) analysis to compare the survival rate of GBPA and other types of GBC to account for variations in cofounding factors; (3) develop and evaluate the price of prognostic nomograms for patients with GBPA.

## Materials and methods

### Data source and data extraction

The SEER database collects cancer incidence and survival data from population-based cancer registries and covers approximately 30% of the cancer population of the United States. The SEER*Stat software version 8.4.0 was used to extract data of patients diagnosed with GBC between 2004 and 2015 from 17 cancer registries available on the SEER database. The primary site label used for the search was C23.9-gallbladder, and the histology codes used were 8140 for GBA, 8840 for GBMA, and 8260 for GBPA. All patients with pathologically confirmed GBC were eligible for this study. However, patients with missing surgical information and an unknown survival or a survival time of less than 1 year were excluded. Since all data in SEER database are anonymous, there is no review by the ethics committee and no informed consent.

### Study variables

The data of this study including; sex (Female, Male), age at diagnosis, marital (Married, Unmarried, Unknown), race (White, Black, Other, Unknown), grade (I, II, II, IV, Unknown), AJCC stage(I, II, II, IV, Unknown), T stage(T1, T2, T3, T4, Unknown), N stage(N0, N1, Unknown), M stage(M0, M1, Unknown), chemotherapy(No/Unknown, Yes), radiation(No/Unknown, Yes), surgery(No, Yes), vital survival status, OS and CSS were extracted for each patient from the SEER database. OS in months was defined as duration from diagnosis to death from any cause. CSS in months was defined as duration from diagnosis to death due to this cancer. The data of patients still alive at the last follow-up were censored.

### Statistical analysis

The categorical variables are shown as numbers with percentages. The Chi-square test was used to compare the categorical variables between the histological GBC groups. Kaplan-Meier survival curves were used to identify the OS and CSS of patients with GBC, and the log-rank test was applied to determine the discrepancy in OS and CSS between GBPA and the other GBC types. Propensity score matching (PSM) analysis was applied to equilibrium the characteristics according to sex, age, marital status, race, tumor grade, AJCC stage, T/N/M stage, and treatment (surgery, radiation, and chemotherapy) and hence minimize the introduction of selection bias when contrasting the OS and CSS among GBC. A p-value below 0.05 was deemed a statistically significant difference in the baseline characteristics, and the clinical variables between the 2 groups were unbalanced. Univariate Cox proportional hazard analysis was used to screening for prognosis factors for OS and CSS in patients with GBPA. The significant influencing factors were included into the multivariate Cox proportional hazard analysis to screening for the independent prognosis factors for OS and CSS. The independent prognosis factors were then used to establish nomograms to predict the OS and CSS in patients with GBPA. The Harrel’s consistency index (C-index) and the area under the curve of ROC were applied to assess the discrimination of the nomograms. Furthermore, in order to check the consistency of the nomograms, calibration curves was used for evaluation. In view of the clinical applicability of the comparison of the nomogram and the standard TNM system, the decision curve analysis (DCA) was used for evaluation. The statistical analyses were executed with the R software (version 4.2.1). It was considered statistically significant when a two-sided p-value below 0.05.

## Results

### Demographic and clinicopathological characteristics

In total, 8282 patients were obtained from the SEER database from 2004 to 2015. From these patients, 1980 were excluded because they did not meet the eligibility criteria. Ultimately, 6302 patients were enrolled in our study, including 281 patients with GBPA, 5798 patients with GBA, and 223 patients with GBMA. The baseline characteristics of the patients with GBPA, GBA and GBMA are presented in **Table 1**. All GBC subtypes were more common in women and patients of white ethnicity. The age and marital status did not differ significantly between GBPA and the other GBC subtypes. However, compared with patients with the GBPA subtype, GBMA was more common in females (P = 0.018), while patients with the GBPA subtype were more likely to be white (P = 0.004).

**Table 1:** Demographic and clinical characteristics of gallbladder carcinoma patients.

Variable	GBPA (n = 281)	GBA (n = 5798)	P value GBPA vs GBA	GBMA (n = 223)	P value GBPA vs GBMA
Sex			0.813		0.018
Male	78 (27.76)	1658 (28.60)		85 (38.12)	
Female	203 (72.24)	4140 (71.40)		138 (61.88)	
Age			0.183		0.277
<60	80 (28.47)	1436 (24.77)		53 (23.77)	
≥60	201 (71.53)	4362 (75.23)		170 (76.23)	
Marital			0.506		0.485
Married	147 (52.31)	2925 (50.45)		105 (47.09)	
Unmarried	120 (42.70)	2643 (45.58)		107 (47.98)	
Unknown	14 (4.98)	230 (3.97)		11 (4.93)	
Race			0.004		0.129
White	192 (68.33)	4428 (76.37)		166 (74.44)	
Black	38 (13.52)	665 (11.47)		31 (13.90)	
Other	51 (18.15)	682 (11.76)		26 (11.66)	
Unknown	0 (0.00)	23 (0.40)		0 (0.00)	
Grade			<0.001		<0.001
I	88 (31.32)	651 (11.23)		35 (15.70)	
II	122 (43.42)	1989 (34.30)		77 (34.53)	
III	30 (10.68)	1715 (29.58)		52 (23.32)	
IV	2 (0.71)	55 (0.95)		3 (1.35)	
Unknown	39 (13.88)	1388 (23.94)		56 (25.11)	
AJCC			<0.001		<0.001
I	202 (71.89)	1628 (28.08)		46 (20.63)	
II	47 (16.73)	1710 (29.49)		73 (32.74)	
III	1 (0.36)	142 (2.45)		7 (3.14)	
IV	22 (7.83)	2052 (35.39)		78 (34.98)	
Unknown	9 (3.20)	266 (4.59)		19 (8.52)	
T			<0.001		<0.001
T1	135 (48.04)	799 (13.78)		24 (10.76)	
T2	106 (37.72)	1726 (29.77)		60 (26.91)	
T3	35 (12.46)	2399 (41.38)		100 (44.84)	
T4	3 (1.07)	324 (5.59)		19 (8.52)	
Unknown	2 (0.71)	550 (9.49)		20 (8.97)	
N			<0.001		<0.001
N0	228 (81.14)	3482 (60.06)		112 (50.22)	
N1	41 (14.59)	1658 (28.60)		74 (33.18)	
Unknown	12 (4.27)	658 (11.35)		37 (16.59)	
M			<0.001		<0.001
M0	256 (91.10)	3550 (61.23)		134 (60.09)	
M1	22 (7.83)	2052 (35.39)		78 (34.98)	
Unknown	3 (1.07)	196 (3.38)		11 (4.93)	
Surgery			<0.001		<0.001
No	6 (2.14)	1570 (27.08)		51 (22.87)	
Yes	275 (97.86)	4228 (72.92)		172 (77.13)	
Radiation			0.223		0.234
No/Unknown	246 (87.54)	4910 (84.68)		186 (83.41)	
Yes	35 (12.46)	888 (15.32)		37 (16.59)	
Chemotherapy			<0.001		<0.001
No/Unknown	222 (79.00)	3441 (59.35)		135 (60.54)	
Yes	59 (21.00)	2357 (40.65)		88 (39.46)	

Patients with the GBPA subtype had a significantly higher rate of low-grade and low AJCC-TNM stage than the GBMA and GBA subtypes (**Table 1**). Patients with the GBPA subtype were significantly more likely to receive surgery (97.86%) than patients with the GBA (72.92%) and GBMA (77.13%) subtypes (P < 0.01 for all). Conversely, patients with GBMA were significantly less likely to receive chemotherapy (79.00%) than patients with the GBA (59.35%) and the GBMA (60.54%) subtypes (P < 0.01 for all).

### Differences in survival between GBPA, GBA, and GBMA

Before PSM analysis, a statistically difference was noted in the OS and CSS between patients with GBPA and all other gallbladder carcinomas, including GBA and GBMA (**Supplementary Figure 1**).

**Figure 1:**
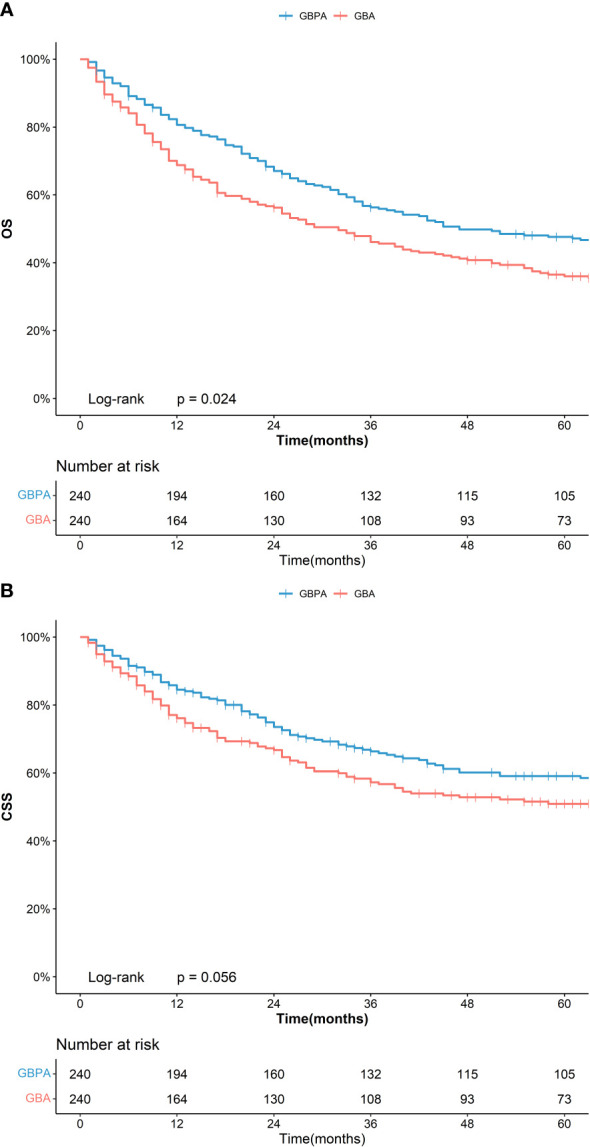
Kaplan–Meier curve for OS **(A)** and CSS **(B)** for GBPA and GBA after PSM.

The PSM analysis revealed no significant difference in the clinical and demographic characteristics between the GBPA, GBA, and GBMA in the three matched cohorts (**Supplementary Table 1**). However, in the matching group with GBPA (n = 240) and GBA (n = 240), the 5-year OS of patients in the GBPA group (47.2%, 95%CI: 41.2%-54.0%) was significantly longer than that of the GBA group (36.0%, 95%CI: 30.2%-42.8%), nevertheless no statistically difference was noted in the 5-year CSS between the GBPA (58.5%, 95%CI: 52.2%-65.6%) and GBA (50.9%, 95%CI: 44.4%-58.4%) groups (**Figure 1**).

**Figure 2:**
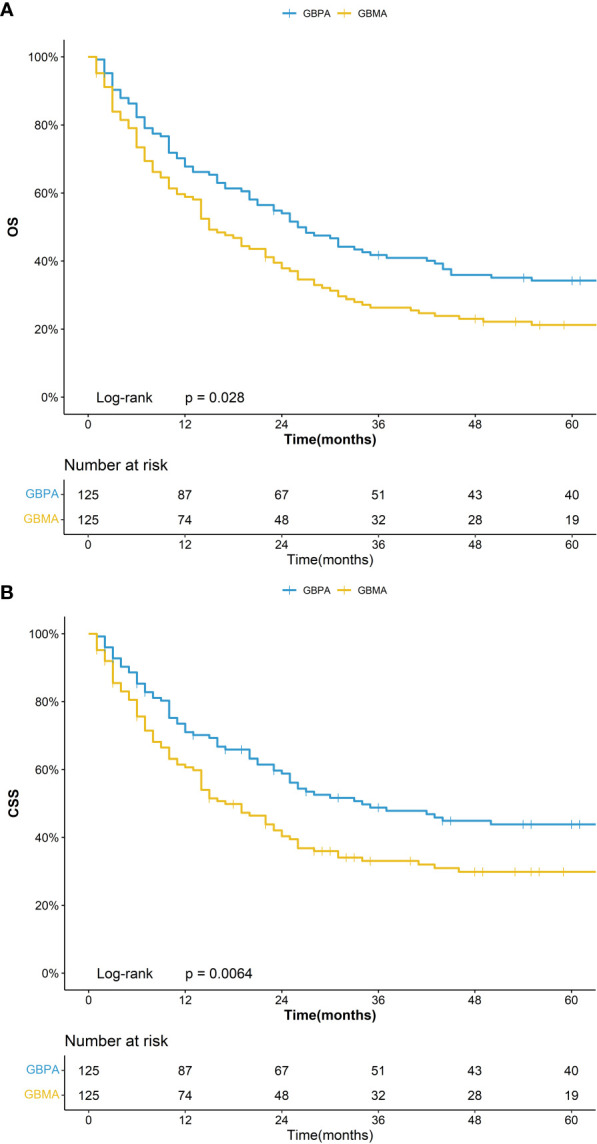
Kaplan–Meier curve for OS **(A)** and CSS **(B)** for GBPA and GBMA after PSM.

In the matching group with GBPA (n = 125) and GBMA (n = 125), the 5-year OS was 34.2% (95%CI: 26.8%-43.8%) for the GBPA group and 21.2% (95%CI: 15.1%-29.9%) for the GBMA group, while the 5-year CSS was 43.8% (95%CI: 35.5%-54.0%) for the GBPA group and 29.8% (95%CI: 22.5%-39.6%) for the GBMA group (**Figure 2**). The differences in the OS and CSS were not statistically significant.

### Screening for prognostic factors for OS and CSS with GBPA

The results for the univariate and multivariate Cox regression analyses are illustrated in **Tables 2**, **3**. Univariate Cox regression model identified age, marital status, T stage, N stage, and M stage as prognostic factors for OS, and T stage, N stage, M stage, surgery, and chemotherapy were identified as prognostic factors for CSS. The multivariate Cox proportional hazard analysis showed that T stage, and M stage were common independent prognostic factors for OS and CSS, In addition, the unique prognostic factors of OS including age and marital, while surgery was the unique prognostic factors of CSS.

**Table 2:** Univariate and multivariate analyses of OS in the cohort.

Characteristics	Univariate COX		Multivariate COX	
	HR (95% CI)	P value	HR (95% CI)	P value
Sex				
Male	Reference			
Female	0.791 (0.561-1.115)	0.180		
Age				
<60	Reference		Reference	
≥60	1.870 (1.264-2.766)	0.002	2.061 (1.379-3.078)	<0.001
Marital				
Married	Reference		Reference	
Unmarried	1.486 (1.088-2.031)	0.013	1.564 (1.135-2.156)	0.006
Race				
White	Reference			
Black	1.209 (0.764-1.914)	0.417		
Other	0.718 (0.465-1.109)	0.136		
T				
T1	Reference		Reference	
T2	1.420 (1.009-1.998)	0.044	1.372 (0.969-1.943)	0.075
T3	3.079 (1.947-4.868)	<0.001	2.471 (1.423-4.289)	0.001
T4	11.501 (1.551-85.296)	0.017	5.071 (0.581-44.26)	0.142
N				
N0	Reference		Reference	
N1	1.968 (1.319-2.936)	0.001	1.355 (0.841-2.184)	0.212
M				
M0	Reference		Reference	
M1	3.237 (1.818-5.764)	<0.001	2.544 (1.334-4.848)	0.005
Surgery				
No	Reference			
Yes	0.433 (0.137-1.361)	0.152		
Radiation				
No/Unknown	Reference			
Yes	0.987 (0.623-1.563)	0.956		
Chemotherapy				
No/Unknown	Reference			
Yes	1.231 (0.841-1.802)	0.285		

**Table 3:** Univariate and multivariate analyses of CSS in the cohort.

Characteristics	Univariate COX		Multivariate COX	
	HR (95% CI)	P value	HR (95% CI)	P value
Sex				
Male	Reference			
Female	0.815 (0.525-1.264)	0.361		
Age				
<60	Reference			
≥60	1.155 (0.741-1.800)	0.526		
Marital				
Married	Reference			
Unmarried	1.159 (0.779-1.724)	0.467		
Race				
White	Reference			
Black	1.487 (0.861-2.569)	0.155		
Other	0.815 (0.472-1.407)	0.462		
T				
T1	Reference		Reference	
T2	1.673 (1.063-2.635)	0.026	1.658 (1.034-2.657)	0.036
T3	4.706 (2.744-8.071)	<0.001	3.333 (1.704-6.518)	<0.001
T4	18.729 (2.469-142.064)	0.005	1.393 (0.105-18.402)	0.801
N				
N0	Reference		Reference	
N1	2.879 (1.833-4.521)	<0.001	1.810 (0.992-3.304)	0.053
M				
M0	Reference		Reference	
M1	3.944 (2.089-7.448)	<0.001	2.262 (1.099-4.656)	0.027
Surgery				
No	Reference		Reference	
Yes	0.233 (0.074-0.738)	0.013	0.221 (0.053-0.920)	0.038
Radiation				
No/Unknown	Reference			
Yes	1.234 (0.722-2.109)	0.442		
Chemotherapy				
No/Unknown	Reference		Reference	
Yes	1.783 (1.154-2.754)	0.009	0.789 (0.445-1.399)	0.417

### Nomograms development for the 1-, 3-, and 5-year OS and CSS

**Figure 3:**
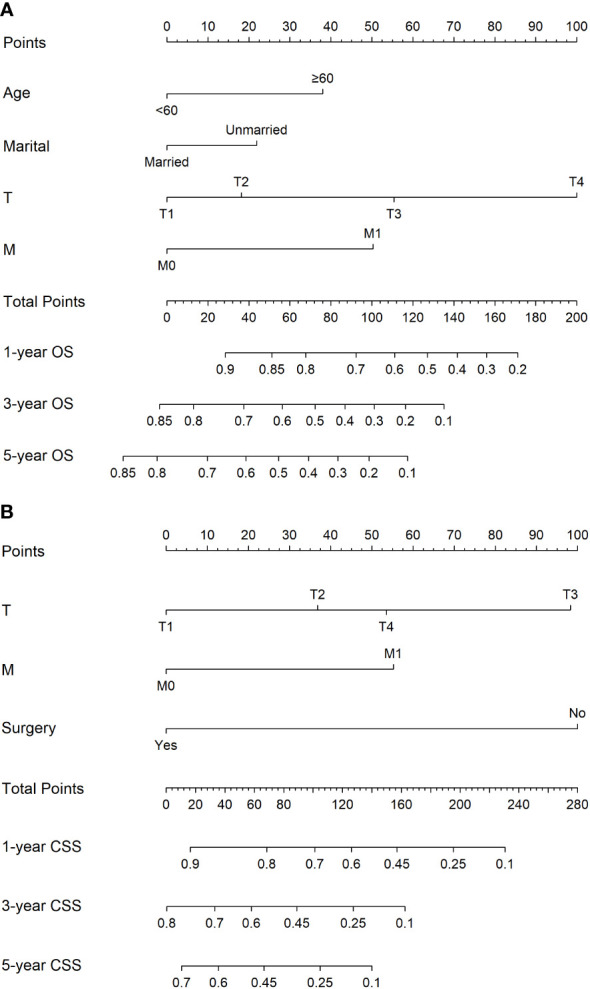
Nomograms for predicting the 1-, 3-, and 5-year OS **(A)** and CSS **(B)**.

The nomograms based on the multivariate Cox regression models for predicting CSS and OS at 1-, 3-, and 5-year in GBPA patients are illustrated in **Figure 3**. Age, marital status, T-stage, and M-stage were identified as the most significant factors influencing OS, and T stage, M stage, and surgery were recognized as the most significant factors influencing CSS in GBPA patients.

### Validation of the nomograms

**Figure 4:**
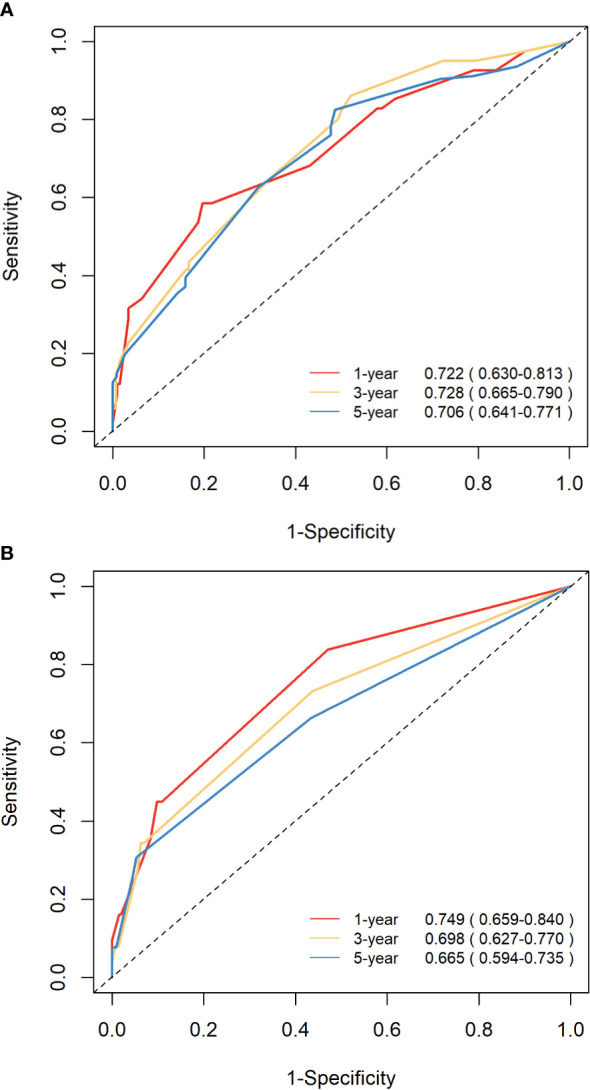
ROC curves demonstrating the discrimination ability of the nomograms in predicting the 1-, 3-, and 5-year OS **(A)** and CSS **(B)**.

**Figure 5:**
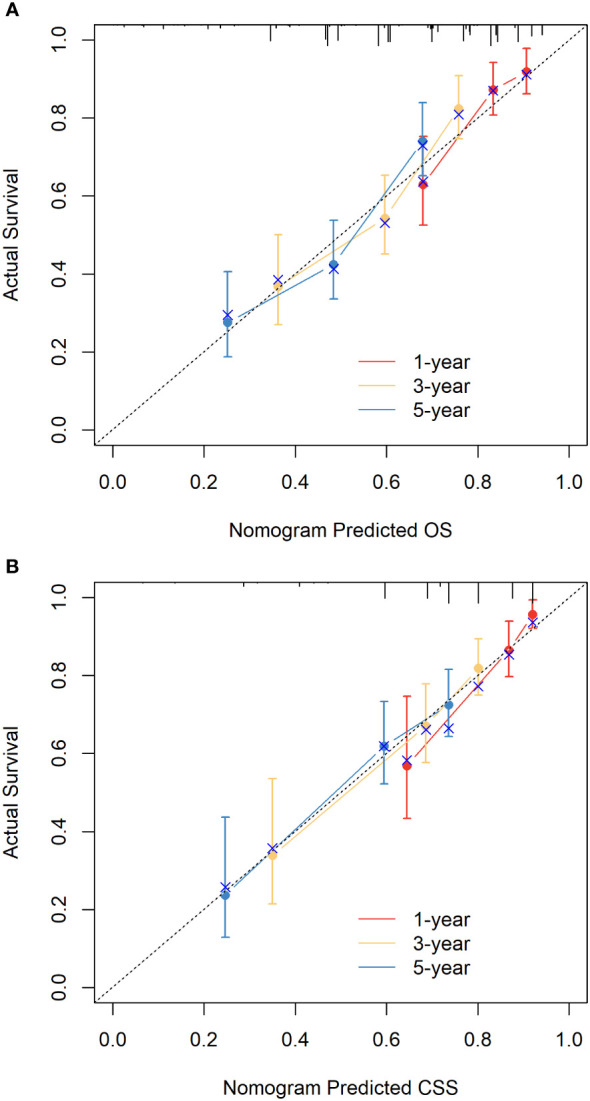
Nomogram calibration curves for the 1-, 3-, and 5-year OS **(A)** and CSS **(B)**.

**Figure 6:**
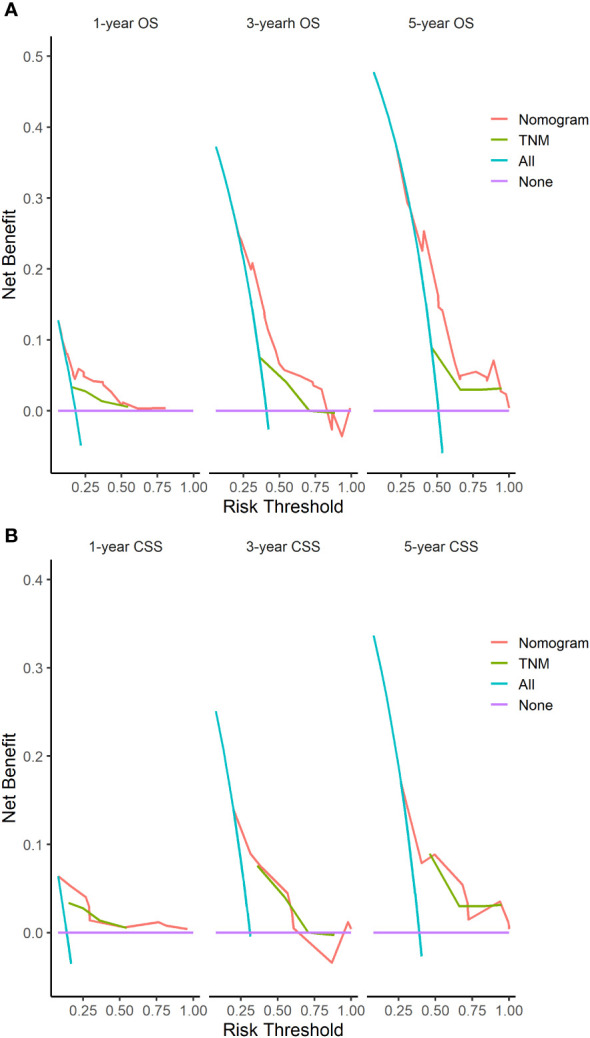
DCA of the nomograms based on the independent variables identified in our study and the TNM staging for the OS **(A)** and CSS **(B)**.

The C-indices of nomograms (OS and CSS) were 0.701 (95% CI: 0.634-0.744) and 0.651 (95%CI: 0.598-0.703), respectively, indicating that the nomograms had a good discrimination ability for predicting OS and CSS. The AUC at 1-, 3-, 5-year were 0.722 (95%CI: 0.630-0.813), 0.728 (95%CI: 0.665-0.790), and 0.706 (95%CI: 0.641-0.771) for OS, the AUC at 1-, 3-, 5-year were 0.749 (95%CI: 0.659-0.840), 0.698 (95%CI: 0.627-0.770), and 0.665 (95%CI: 0.594-0.735) for CSS, respectively (**Figure 4**). The calibration curve based on the bootstrap resampling validation of the nomograms (OS and CSS) performed well in predicting the probability. (**Figure 5**). From the perspective of clinical applicability, the DCA analysis showed that the nomograms had an effective net benefit and were preferred at predicting OS and CSS than the traditional TNM system (**Figure 6**).

## Discussion

In 2020, there will be an estimated 115949 new GBC cases worldwide, ranking 25th out of 36 cancers in 185 countries (17). GBPA is a rare subtype of GBC. Due to its low incidence rate (14), the OS and CSS are still unclear as large sample cohort studies are lacking (9, 10). As a result, further research is required to evaluate the clinical significance of this GBC subtype. To our knowledge, there is no relevant literature comparing survival rates (OS and CSS) of GBPA with other GBC subtypes after PSM. Therefore, the first population-based large-sample research aiming to build nomograms to predict survival rates (OS and CSS) of GBPA based on clinical, demographic, and outcome data obtained from the SEER database.

Previous studies have shown that GBC is the only cancer in the digestive system that is more common in women than men (18, 19). Consistent with previous studies, we also found that all GBC subtypes are more common in women. However, the ratio of female of GBPA was significantly higher than that of GBMA. Similar to previous studies, more than 70% of the patients with the GBC subtypes were aged 60 years and above (20). Compared with GBA, the GBPA subtype was less common in white patients, while no significant racial differences were noted between the GBPA and GBMA subtypes.

Studies have also shown that GPBA has unique pathological characteristics. Consistent with our findings, Wan et al. (10) also showed that the differentiation degree and TNM stage between the GBPA and GBA subtypes differed. Zou et al. (21) also showed a difference in the TNM staging between GBA patients and GBMA patients, but no comparison was made between GBPA and GBMA patients.

One of the previous research (10) have also confirmed that the prognosis of GBPA is better than other types of GBC and the 1-year, 3-year, 5-year, and 10-year OS of patients with GBPA were 100%, 76.9%, 76.9%, and 38.5%, respectively. However, the study based on Chinese data has been almost 10 years old and the sample of patients with GBPA is only 16. There were 281 GBPA patients in our study and showed that the 1-year, 3-year, 5-year, and 10-year OS of patients with GBPA were 81.3%, 58.8%, 51.7%, and 34.2% before PSM, while the 1-year, 3-year, 5-year, and 10-year OS of patients with GBPA were 80.6%, 56.3%, 47.2%, and 33.3% after PSM. Our research results have a sufficiently large sample and comparative analysis before and after PSM, which to some extent avoids bias in the study, so the results obtained may be more reliable.

In this study, PSM analysis (ratio 1:1) was used to represent survival status differences between GBPA and other forms of GBC (GBA and GBMA). In line with previous reports (14, 16, 18, 22, 23), the data before PSM indicated that patients with the GBPA subtype had a higher OS and CSS than patients with the GBA and GBMA subtypes. However, after applying PSM, GBPA still had a higher OS than GBA, but no statistical difference was noted in the CSS. Meanwhile, the OS and CSS of GBPA and GBMA remained significantly different.

The prognosis of patients with GBPA is relatively good, which may be related to the inherent characteristics of this tumor. Compared with other types of GBC, the ability of GBPA to infiltrate the gallbladder wall is reduced, which to some extent delays the progression of the disease. In addition, Zou et al. (10) have shown that obstructive symptoms related to GBPA lesions may occur earlier, which helps early detection and treatment of GBPA patients and to some extent, improves the prognosis of GBPA patients.

Cox proportional hazard analysis was used to determine prognostic factors in patients with GBPA. Age, marital, T stage, and M stage were recognized as independent prognosis factors of OS in GBPA patients, while T-stage, M-stage, and surgery were recognized as independent prognosis factors of CSS in GBPA patients. These findings are consistent with the study of Wan et al. (10), which showed that jaundice, T-stage, and nodal involvement were independent predictors for OS in patients with GBPA. More than 90% of GBPA patients in this study had no distant metastasis. As a result, most of the GBPA patients (97%) in our study were treated with surgery. Previous reports (24–26) have shown that surgery is an effective treatment for GBC, but there are some controversies about the prognosis. Our findings indicate that surgical treatment in GBPA patients can improve CSS but not the OS. Conversely, chemotherapy and radiotherapy were not identified as independent prognosis factors for OS and CSS in patients with GBPA.

The independent prognosis factors were used to establish predictive nomograms (OS and CSS), specifically for patients with the GBPA subtype. The ROC curve and calibration chart showed that our proposed nomogram has good prediction ability. Furthermore, the DCA showed that the application of this nomogram in clinical practice provided a positive net clinical benefit compared to the standard TNM staging system.

Our study has some limitations that have to be acknowledged. Owing to a population-based retrospective analysis based on data available on the SEER database, important variables, such as preoperative laboratory results and comorbidities, could not be analyzed and incorporated into our prediction model. Finally, all the data in our study were obtained from a single open-source database. Therefore before applying this model clinically, external validation is required.

## Conclusions

In the study, we carried out a population-based study using the SEER database to demonstrate the clinicopathological features and survival of patients with GBPA. The results demonstrated that GBPA is an unusual GBC with unique characteristics and prognosis. In addition, we have also establishmented nomograms to predict survival rates (OS and CSS) of GBPA patients. The established nomograms provided a better survival prediction for GBPA patients than the traditional TNM model. The nomograms could be used to guide clinicians in conducting personalized diagnosis and treatment of GBPA patients. However, further validation of external data is needed to assess the generalizability of the findings.

## Data availability statement

The original contributions presented in the study are included in the article/**Supplementary Material**. Further inquiries can be directed to the corresponding author.

## Author contributions

ZW, HW and LW designed and performed the study. HW, XZ provided administrative support. ZW, LW, YH and XZ collected and assembled the data. YB, ZW contributed to data processing. ZW, LW and YB prepared all the tables and figures, interpretation of results, and drafting. All authors reviewed and approved the manuscript. All authors contributed to the article and approved the submitted version.

## Supplementary material

Supplementary Figure 1 Kaplan–Meier curves for OS **(A)** and CSS **(B)** for the GBPA and GBA subtypes before PSM. Kaplan–Meier curves for OS **(C)** and CSS **(D)** for the GBPA and GBMA before PSM.

Supplementary Table 1 Demographic and clinical characteristics of gallbladder carcinoma patients after PSM
