# Supplementary figures and images for: Development and validation of a prognostic nomogram for gallbladder papillary adenocarcinoma
Source: Front Oncol. 2023 May 16;13:1157057. doi: 10.3389/fonc.2023.1157057 (PMC10228726; doi:10.3389/fonc.2023.1157057)

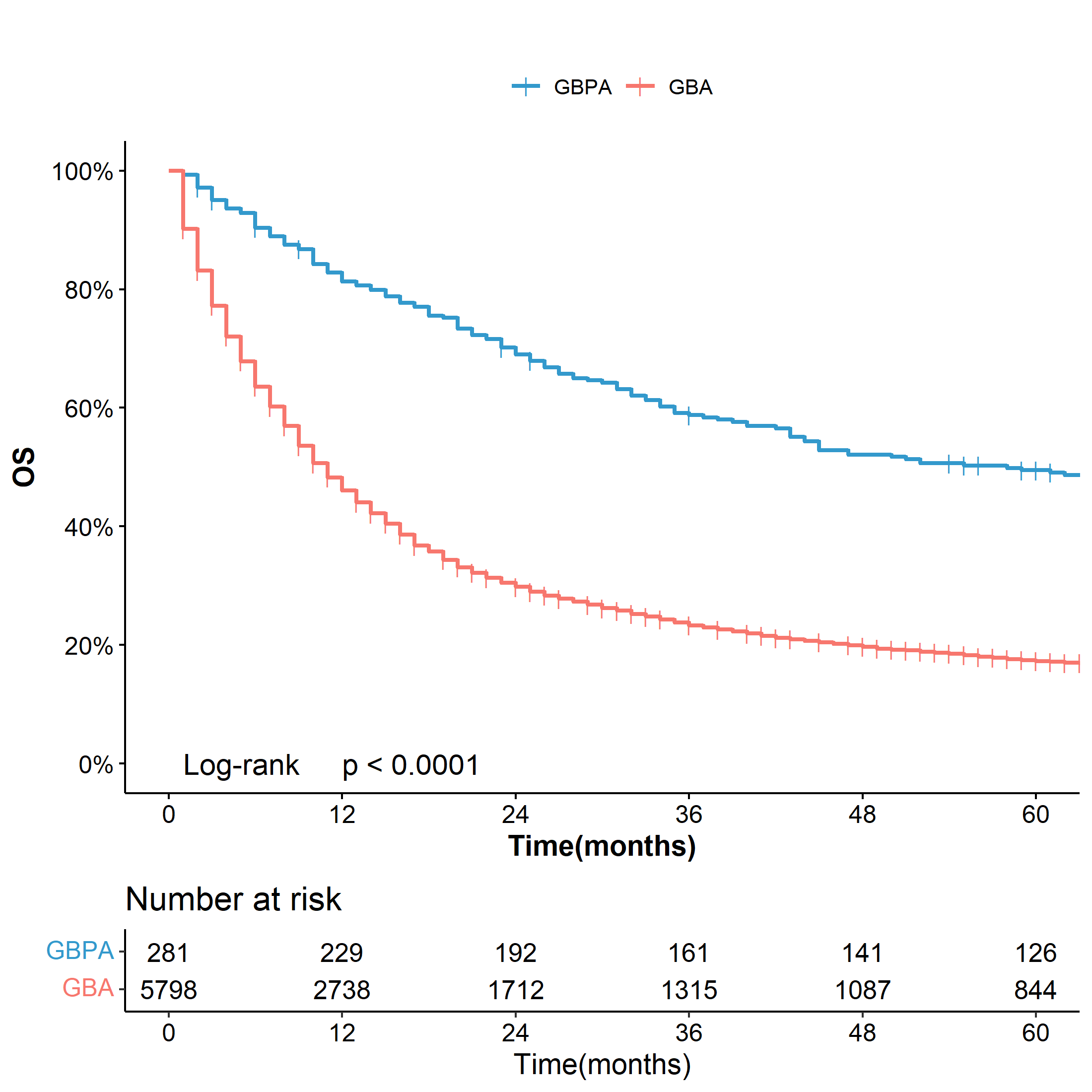

Supplement: Supplementary Figure 1 — Kaplan–Meier curves for OS (A) and CSS (B) for the GBPA and GBA subtypes before PSM. Kaplan–Meier curves for OS (C) and CSS (D) for the GBPA and GBMA before PSM. [file Image_1.tiff]

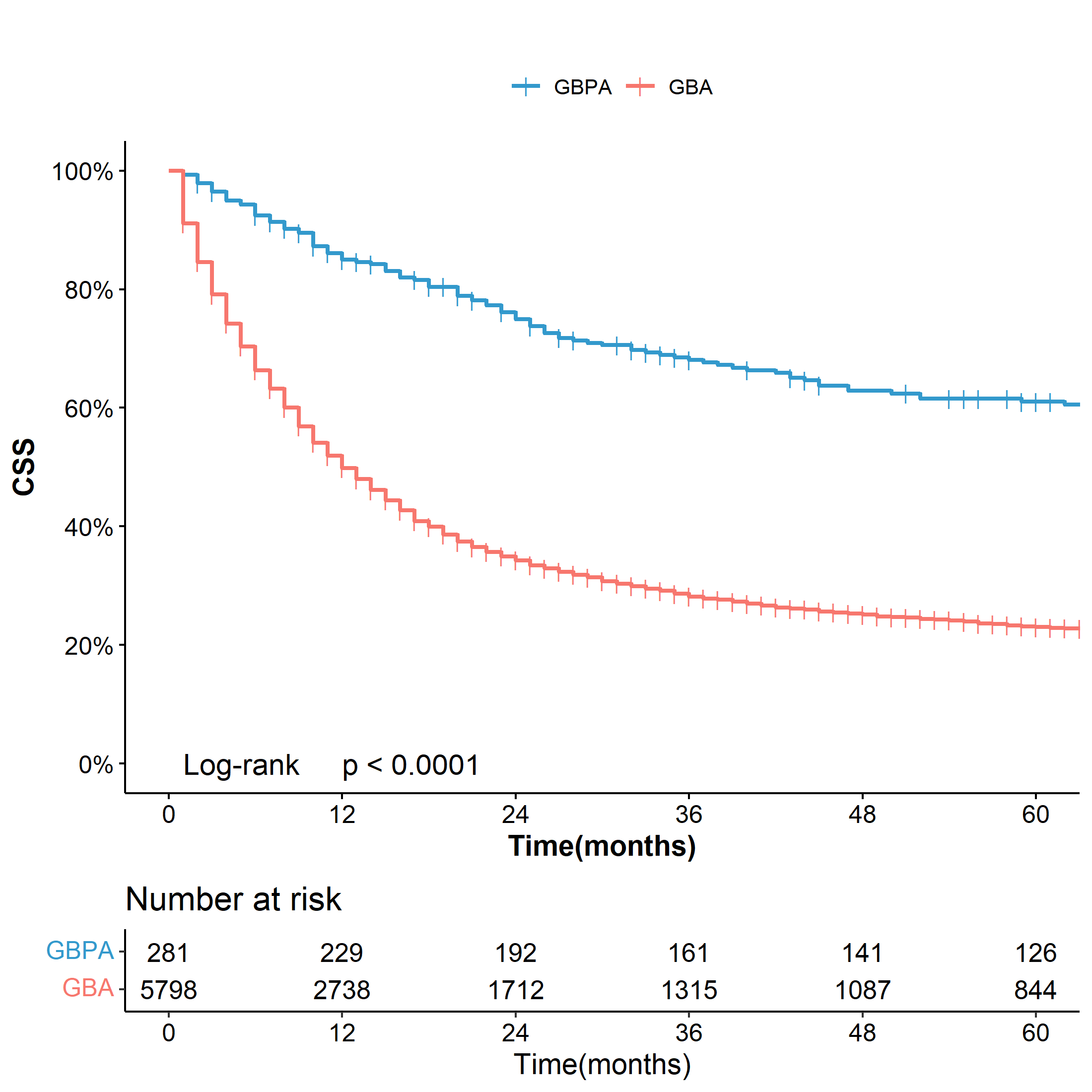

Supplement: Supplementary Table 1 — Demographic and clinical characteristics of gallbladder carcinoma patients after PSM [file Image_2.tiff]

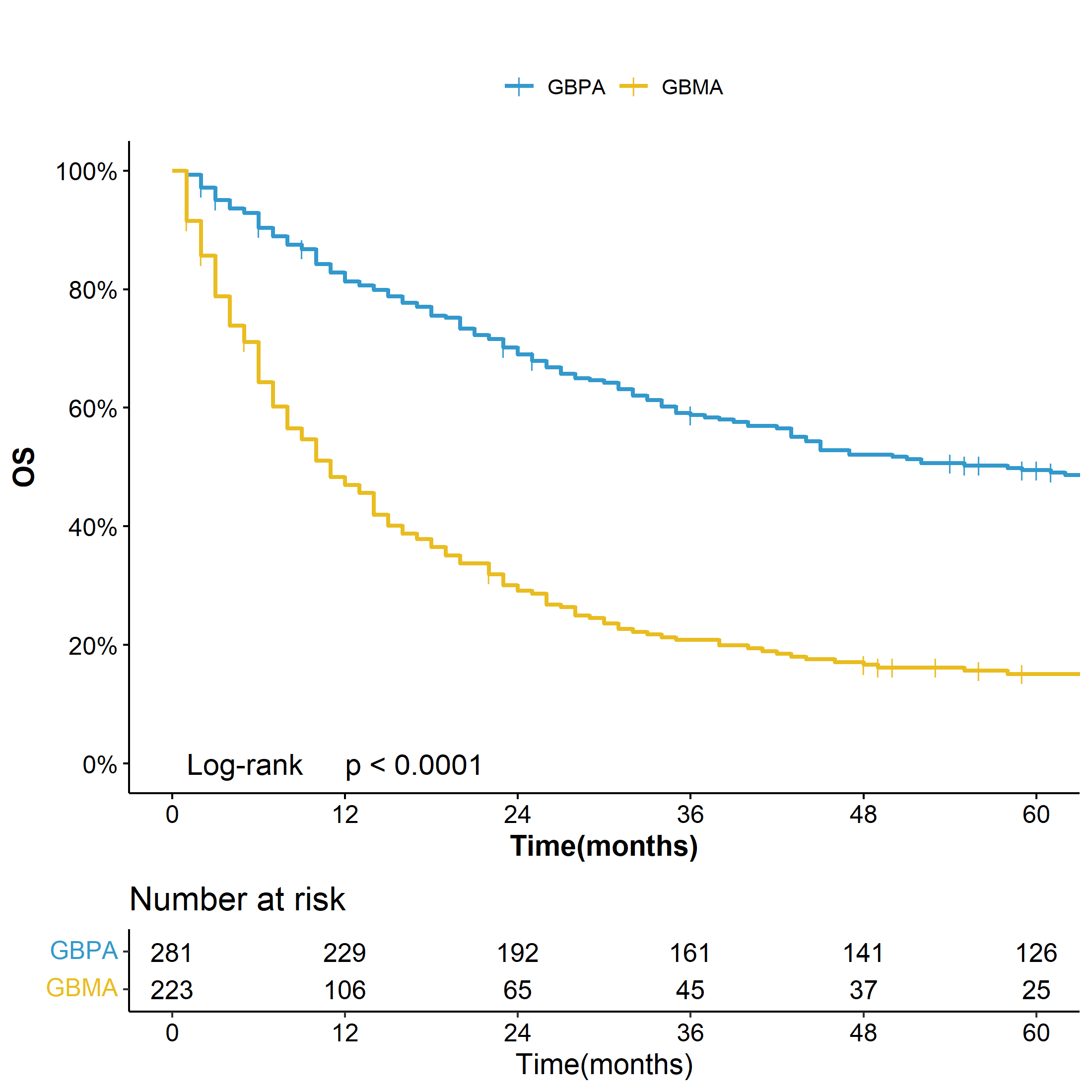

Supplement: Supplementary file 3 [file Image_3.tiff]

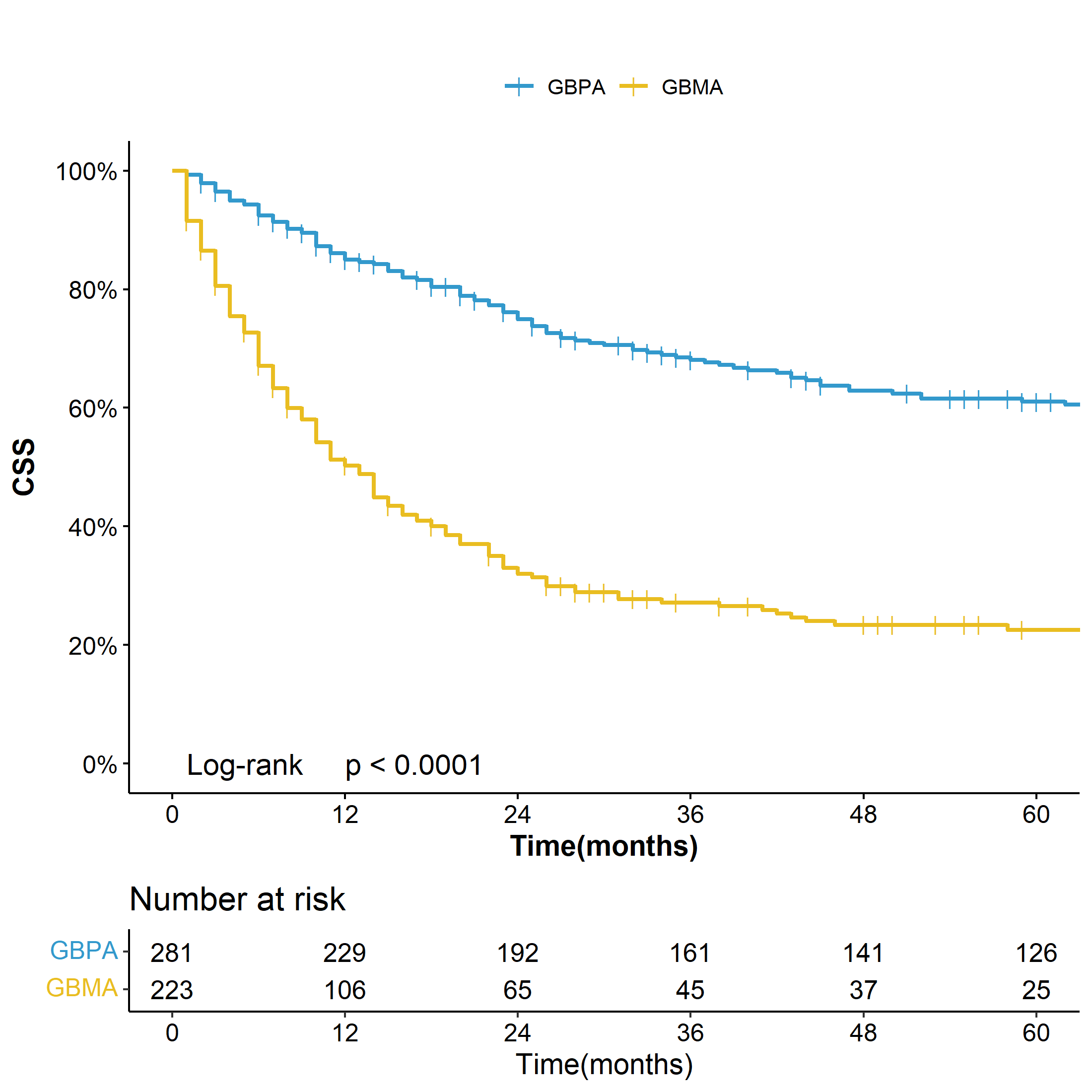

Supplement: Supplementary file 4 [file Image_4.tiff]
