# Supplementary material for: Development and validation of a prognostic nomogram for gallbladder papillary adenocarcinoma
Source: Front Oncol. 2023 May 16;13:1157057. doi: 10.3389/fonc.2023.1157057 (PMC10228726; doi:10.3389/fonc.2023.1157057)
Supplement: Supplementary file 5 [file Table_1.docx]

### Supplementary Table 1：Demographic and clinical characteristics of gallbladder carcinoma patients after PSM

| **Variable** | **GBPA (n = 240)** | **GBA (n = 240)** | **P value GBPA vs. GBA** | **GBPA (n = 125)** | **GBMA (n = 125)** | **P value GBPA vs. GBMA** |
| --- | --- | --- | --- | --- | --- | --- |
| Sex |  |  | 0.999 |  |  | 0.288 |
| Male | 62 (25.83) | 63 (26.25) |  | 39 (31.20) | 48 (38.40) |  |
| Female | 178 (74.17) | 177 (73.75) |  | 86 (68.80) | 77 (61.60) |  |
| Age |  |  | 0.831 |  |  | 0.999 |
| <60 | 56 (23.33) | 59 (24.58) |  | 29 (23.20) | 30 (24.00) |  |
| ≥60 | 184 (76.67) | 181 (75.42) |  | 96 (76.80) | 95 (76.00) |  |
| Marital |  |  | 0.474 |  |  | 0.738 |
| Married | 128 (53.33) | 120 (50.00) |  | 66 (52.80) | 60 (48.00) |  |
| Unmarried | 104 (43.33) | 107 (44.58) |  | 53 (42.40) | 59 (47.20) |  |
| Unknown | 8 ( 3.33) | 13 ( 5.42) |  | 6 ( 4.80) | 6 ( 4.80) |  |
| Race |  |  | 0.910 |  |  | 0.425 |
| White | 177 (73.75) | 175 (72.92) |  | 85 (68.00) | 93 (74.40) |  |
| Black | 26 (10.83) | 29 (12.08) |  | 18 (14.40) | 17 (13.60) |  |
| Other | 37 (15.42) | 36 (15.00) |  | 22 (17.60) | 15 (12.00) |  |
| Grade |  |  | 0.672 |  |  | 0.743 |
| I | 68 (28.33) | 65 (27.08) |  | 30 (24.00) | 25 (20.00) |  |
| II | 110 (45.83) | 99 (41.25) |  | 55 (44.00) | 50 (40.00) |  |
| III | 28 (11.67) | 31 (12.92) |  | 20 (16.00) | 25 (20.00) |  |
| IV | 2 ( 0.83) | 3 ( 1.25) |  | 1 ( 0.80) | 2 ( 1.60) |  |
| Unknown | 32 (13.33) | 42 (17.50) |  | 19 (15.20) | 23 (18.40) |  |
| AJCC |  |  | 0.664 |  |  | 0.723 |
| I | 176 (73.33) | 169 (70.42) |  | 52 (41.60) | 44 (35.20) |  |
| II | 42 (17.50) | 39 (16.25) |  | 42 (33.60) | 43 (34.40) |  |
| III | 1 ( 0.42) | 1 ( 0.42) |  | 1 ( 0.80) | 2 ( 1.60) |  |
| IV | 18 ( 7.50) | 25 (10.42) |  | 21 (16.80) | 28 (22.40) |  |
| Unknown | 3 ( 1.25) | 6 ( 2.50) |  | 9 ( 7.20) | 8 ( 6.40) |  |
| T |  |  | 0.530 |  |  | 0.461 |
| T1 | 111 (46.25) | 122 (50.83) |  | 21 (16.80) | 24 (19.20) |  |
| T2 | 92 (38.33) | 86 (35.83) |  | 64 (51.20) | 50 (40.00) |  |
| T3 | 33 (13.75) | 26 (10.83) |  | 35 (28.00) | 44 (35.20) |  |
| T4 | 2 ( 0.83) | 5 ( 2.08) |  | 3 ( 2.40) | 3 ( 2.40) |  |
| Unknown | 2 ( 0.83) | 1 ( 0.42) |  | 2 ( 1.60) | 4 ( 3.20) |  |
| N |  |  | 0.216 |  |  | 0.893 |
| N0 | 197 (82.08) | 189 (78.75) |  | 77 (61.60) | 74 (59.20) |  |
| N1 | 38 (15.83) | 39 (16.25) |  | 36 (28.80) | 37 (29.60) |  |
| Unknown | 5 ( 2.08) | 12 ( 5.00) |  | 12 ( 9.60) | 14 (11.20) |  |
| M |  |  | 0.205 |  |  | 0.501 |
| M0 | 221 (92.08) | 211 (87.92) |  | 101 (80.80) | 95 (76.00) |  |
| M1 | 18 ( 7.50) | 25 (10.42) |  | 21 (16.80) | 28 (22.40) |  |
| Unknown | 1 ( 0.42) | 4 ( 1.67) |  | 3 ( 2.40) | 2 ( 1.60) |  |
| Surgery |  |  | 0.600 |  |  | 0.221 |
| No | 6 ( 2.50) | 9 ( 3.75) |  | 6 ( 4.80) | 12 ( 9.60) |  |
| Yes | 234 (97.50) | 231 (96.25) |  | 119 (95.20) | 113 (90.40) |  |
| Radiation |  |  | 0.259 |  |  | 0.872 |
| No/Unknown | 216 (90.00) | 207 (86.25) |  | 102 (81.60) | 100 (80.00) |  |
| Yes | 24 (10.00) | 33 (13.75) |  | 23 (18.40) | 25 (20.00) |  |
| Chemotherapy |  |  | 0.144 |  |  | 0.506 |
| No/Unknown | 197 (82.08) | 183 (76.25) |  | 85 (68.00) | 79 (63.20) |  |
| Yes | 43 (17.92) | 57 (23.75) |  | 40 (32.00) | 46 (36.80) |  |
